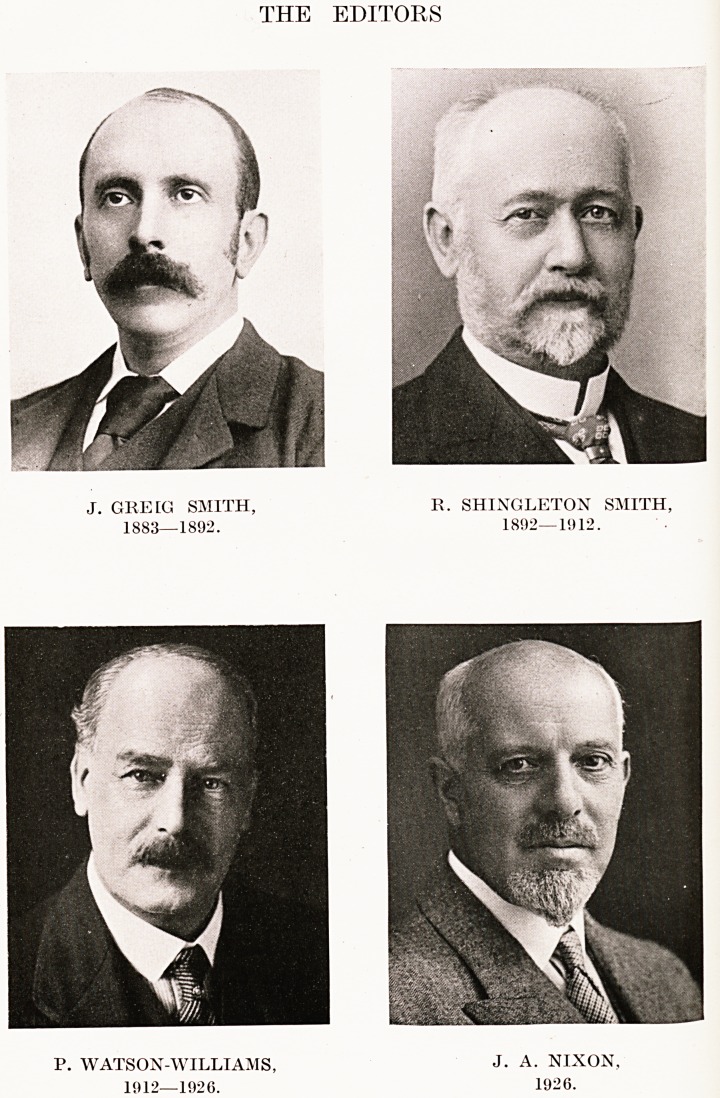# Editorial Note

**Published:** 1933

**Authors:** 


### THE EDITORS

J. GREIG SMITH,
1883—1892.

R. SHINGLETON SMITH,

1892—1912.

P. WATSON-WILLIAMS,
1912—1926.

J. A. NIXON,
1926.

### The Bristol Medico-Chirurgical Journal

*"Scire est nescire, nisi id me
Scire alius sciret."*

AUTUMN, 1933.

### EDITORIAL NOTE.

The coincidence of the centenary of the Medical School with the completion of the fiftieth volume of this *Journal* has been made the occasion for the issue of a special number. It contains a description of the centenary celebrations at the University, together with a survey of the *Journal* during its unbroken existence of fifty years. The opportunity has also been taken of looking back over a longer past and rescuing from oblivion some pioneers in medicine as well as reviving in the memories of our readers some unforgotten names. In the list of contributors in Bristol to the progress of medicine only those who have been indisputably first in the field are included, and the limits of birth or residence have been strictly observed. No mention has been made of any medical man now practising. The history of the medical school with lists of its alumni compiled and written by Dr. G. Parker appeared at the time of the centenary

N

celebrations. There is no need to go again over the ground which he has so admirably covered. But we feel that this is a suitable opportunity of sending special messages of congratulation to three grand veterans of the Bristol Medical School, Conrad Fitzgerald (M.R.C.S., L.S.A., 1870) and John Edward Shaw (M.B., B.S. Edin., 1872), who entered the School in 1866, and Henry Waldo (M.D. Aberd., 1873, M.R.C.P. Lond., 1888), who entered in 1867. Dr. Fitzgerald lives at Fortune Bay, Newfoundland. Drs. Shaw and Waldo are Consulting Physicians to the Bristol Royal Infirmary and reside in Clifton. Their connection with the School covers two-thirds of its total existence. The history of the *Journal* has been written by Dr. P. Watson-Williams, whose intimate acquaintance with its initiation and progress enables him to describe events from first-hand knowledge rather than from studying its volumes and minutes alone. Bristol has been fortunate in having possessed so many medical chroniclers of contemporary events in the past, thereby the gap that usually divides the present from the past is less noticeable here than elsewhere. The habit of keeping green the memories of great men is due in a large measure to the labours of Richard Smith, the indefatigable surgeon who left behind him fifteen volumes of MS. records carefully preserved in the Royal Infirmary. Our aim in this *Journal* since its foundation has been to speak of the moderns without contempt and of the ancients without idolatry, believing that a knowledge of great traditions is the foundation of high hopes and a pride in past achievement is an encouragement to progress.

An index to the first fifty volumes of the *Journal* is being compiled, and will be issued to all subscribers with our next number.

## Figures and Tables

**Figure f1:**